# Cell junctions and oral health

**DOI:** 10.17179/excli2019-1370

**Published:** 2019-06-07

**Authors:** Mohammad Samiei, Elham Ahmadian, Aziz Eftekhari, Mohammad Ali Eghbal, Fereshte Rezaie, Mathieu Vinken

**Affiliations:** 1 Faculty of Dentistry, Tabriz University of Medical Sciences, Tabriz, Iran; 2 Dental and Periodontal Research center, Tabriz University of Medical Sciences, Tabriz, Iran; 3 Students Research Committee, Tabriz University of Medical Sciences, Tabriz, Iran; 4 Pharmacology and Toxicology department, Maragheh University of Medical Sciences, Maragheh, Iran; 5 Drug Applied Research Center and Pharmacology and Toxicology department, Tabriz University of Medical Sciences, Tabriz, Iran; 6 General Practitioner, Tabriz University of Medical Sciences, Tabriz, Iran; 7 Department of In Vitro Toxicology and Dermato-Cosmetology, Vrije Universiteit Brussel, Brussels, Belgium

**Keywords:** tight junction, gap junction, anchoring junction, oral health, oral disease

## Abstract

The oral cavity and its appendices are exposed to considerable environmental and mechanical stress. Cell junctions play a pivotal role in this context. Among those, gap junctions permit the exchange of compounds between cells, thereby controlling processes such as cell growth and differentiation. Tight junctions restrict paracellular transportation and inhibit movement of integral membrane proteins between the different plasma membrane poles. Adherens junctions attach cells one to another and provide a solid backbone for resisting to mechanistical stress. The integrity of oral mucosa, normal tooth development and saliva secretion depend on the proper function of all these types of cell junctions. Furthermore, deregulation of junctional proteins and/or mutations in their genes can alter tissue functioning and may result in various human disorders, including dental and periodontal problems, salivary gland malfunction, hereditary and infectious diseases as well as tumorigenesis. The present manuscript reviews the role of cell junctions in the (patho)physiology of the oral cavity and its appendices, including salivary glands.

## Introduction

Cell junctions, as crucial coordinators of several processes such as cell growth, differentiation, morphogenesis and tissue repair, play diverse roles in many aspects of tissue homeostasis (Khalili and Ahmad, 2015[40]). They generate physical and molecular asymmetry in cell surfaces, which leads to the establishment of apical and basolateral membrane poles that are pivotal in tissue development (Rehder et al., 2006[63]; Shin et al., 2006[72]). The presence of various cell-cell and cell-extracellular matrix junctions enables epithelial cells to secure tight connections with a sustained polarity (Wan et al., 2018[78]). In the oral cavity, the stratified squamous epithelium in the outmost layer of the oral mucous membrane protects against microbial infections and mechanical stress (Groeger and Meyle, 2015[31]). Highly specific junctional complexes between these cells maintain the integrity of the mucosal barrier. The dynamic properties of cell junctions facilitate their rapid rearrangements in physiological and pathological conditions. Cell junctions in epithelia are classified into 3 groups known as gap junctions (GJs), tight junctions (TJs) and anchoring junctions. Anchoring junctions are further subclassified as adherens junctions (AJs), desmosomes and hemi-desmosomes (Bosveld et al., 2016[10]). The current manuscript focuses on the role of cell junctions in the oral cavity. 

## Tight Junctions

### General features of tight junctions

**Figure 1:**
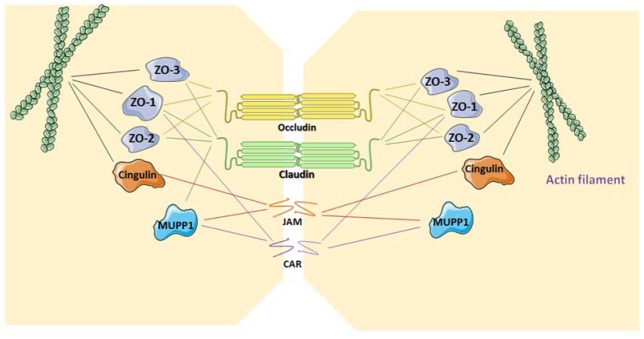
Structure of tight junctions. The transmembrane proteins claudin and occludin, bind to intracellular zonula occludens (ZO) proteins. Junctional adhesion molecules (JAM)/coxsackievirus and adenovirus receptor (CAR), as transmembrane glycoproteins, bind to cingulin and/or multi-PDZ domain protein 1 (MUPP1). CAR is also attached to ZO-1. ZO proteins and cingulin link the tight junction structure to the actin filament.

TJs form the closest cell-cell interactions in the apical area of epithelial and endothelial cells (Figure 1(Fig. 1)). TJs occur in tissues that display polarized secretory and absorptive activity, such as oral mucosa and the intestine, and have 2 main functions. The first function is the so-called gate function that generates the permeability barrier between neighboring cells and that controls the flux of ions and non-electrolytes through the paracellular space (Matter and Balda, 2003[54]). The second function is known as the fence function and permits cells to act in a polarized manner, which is crucial in morphogenesis, protein-membrane trafficking and transportation. This is accomplished through the formation of a boundary nearby the apex lateral plasma membrane that prevents the exchange of proteins and lipids between the 2 membrane areas. Upon transmission electron microscopy, TJs appear as sealing points where the external leaflets of adjacent membranes are tightly apposed (Wan et al., 2018[78]).

TJs are formed by a number of transmembrane proteins, including occludin, claudin, junctional adhesion molecules (JAM)/coxsackievirus and adenovirus receptor (CAR), as well as cytoplasmic molecules, such as zonula occludens (ZO), cingulin and multi-PDZ domain protein 1 (MUPP1), which are linked to the actin cytoskeleton (Table 1(Tab. 1)) (Leech et al., 2015[45]). Structurally, claudin and occludin belong to a family of proteins that have 4 transmembrane domains and 2 extracellular loops, whereas JAM and CAR have immunoglobulin-like domains and are classified as type 1 transmembrane glycoproteins. The localization of occludin in TJs occurs upon its phosphorylation and binding to ZO-1 and ZO-3 (Sakakibara et al., 1997[68]). However, studies with knock-out mice demonstrate that occludin is not strictly necessary for the generation of TJs. Moreover, occludin-deficient epithelial cells show a normal TJ pattern (Baker, 2010[6]). On the contrary, claudins are indispensable TJ proteins in terms of forming epithelial barriers and transportation systems (Markov et al., 2015[50]). The highly conserved PDZ-binding motifs in the C-terminus of claudins bind to ZO-1, ZO2 and ZO-3. Claudins are divided into 3 classes according to their function, namely claudins with sealing activity, claudins that enable paracellular permeability and claudins with ambiguous functionality (Leech et al., 2015[45]). ZO-1 is the major cytoplasmic protein of TJs and binds to claudins and occludin (Anderson, 2001[5]). Occludin, which establishes a permeable barrier, is the main transmembrane protein found at the sealing strands of TJs. While the transmembrane proteins form sealing or intercellular barriers between adjacent cells, the cytoplasmic proteins provide intracellular connections for TJs. The immunoglobulin-like surface molecules JAM and CAR participate in cell adhesion and intracellular recognition, and are located in regions of cell-cell contacts. JAM proteins are divided into 5 sub-families and are expressed in epithelial and endothelial cells as well as in blood cells (Mandell and Parkos, 2005[49]). The PDZ-binding motif in the C-terminus of JAM binds to cingulin and MUPP1. CAR, which was first identified as a protein related to coxsackieviruses and that was later characterized as an adenovirus receptor, binds to the PDZ domain of ZO-1 and MUPP1 (Coyne and Bergelson, 2005[16]). 

### Tight junctions and oral health

A number of studies have investigated TJs in the oral mucosa. Salivary glands have been found to express some TJ proteins, such as occludin, claudins and ZO-1 (Baker, 2010[6]). The acinar cells and intercalated ducts express claudin-3, while the striated and interlobular ducts show the production of claudin-4 in rat salivary glands (Peppi and Ghabriel, 2004[61]). Furthermore, claudin-4 was detected at all developmental stages in mouse salivary glands. Occludin is abundantly present in the duct system (Peppi and Ghabriel, 2004[61]). Saliva production is a critical element in basic functions of the oral cavity, including swallowing and digestion, and exerts anti-microbial activity. The primary constituents of saliva, namely ions, electrolytes, proteins, and lipids, are secreted from acinar cells of major salivary glands and become modified in salivary ducts (Carpenter, 2013[13]). Upon the release of acetylcholine, parasympathetic nerves stimulate fluid and electrolyte secretion, whereas protein secretion is mediated *via* sympathetic nerves (Masedunskas et al., 2011[52]). In particular, the muscarinic action of acetylcholine leads to accumulation of free calcium inside cells, which subsequently increases the efflux of chloride and potassium (Catalán et al., 2009[14]). This promotes the activity of the water channel aquaporin-5, which enhances transcellular water secretion as well as the activity of TJs to control paracellular sodium and water diffusion (Kawedia et al., 2007[39]). TJs play a major role in both water secretion and secretory granule exocytosis in salivary secretion. The molecular composition of TJs affects paracellular permeability during water secretion (Rosenthal et al., 2010[65]). Moreover, water transportation through TJs is diminished by the lack of aquaporin-5. The recruitment of F-actin and non-muscle myosin II by salivary glands produces the driving force required to complete the collapse of secretory granules during exocytosis (Masedunskas et al., 2011[52]). Thus, alterations in the organization and composition of TJs mediate water secretion and secretory granule exocytosis in saliva. 

Sjӧgren's syndrome (SS) is an auto-immune secretory disorder and is clinically manifested as a chronic inflammatory condition in salivary and lacrimal glands, resulting in a progressive decline in the secretion of saliva and tears, and hence dryness in the mouth and eyes (Maślińska et al., 2015[53]). Acinar damage, extensive lymphocytic infiltration and local release of inflammatory cytokines, such as interleukin (IL)-6, IL-1β, IL-10, interferon-γ (IFN-γ) and tumor necrosis factor-α (TNF-α), are typical features of SS (Fox et al., 1994[25]). The organization and expression of TJ proteins drastically change in salivary glands in SS. In this regard, occludin and ZO-1 are downregulated, whereas claudin-1 and claudin-4 are overexpressed in salivary glands of SS patients. In addition, these claudins move from the apical to the basolateral side of acinar cells in minor salivary glands (Ewert et al., 2010[23]). The release of pro-inflammatory cytokines disrupts the integrity of TJs. It has been shown that IFN-γ and TNF-α compromise the barrier function of TJs, which is associated with a drop in claudin-1 production (Baker et al., 2008[7]). Thus, the disruption of TJ integrity in SS is closely linked to the secretion of inflammatory mediators. 

Tissue development, architecture and function strongly rely on the structure and function of cell-cell junctions. The reciprocal interaction between oral epithelium and neural crest-mesenchyme supports the generation of teeth. Epithelial thickening, as the initial sign of tooth development, is followed by differentiation (Palacios et al., 2004[60]). While mesenchymal cells or dental papilla form dentin-secreting odontoblasts, epithelial cells turn into enamel-secreting ameloblasts. Morphogenesis and cell differentiation in tooth germ cells are affected by cell-cell junctions. All types of cell-cell and cell-extracellular matrix junctions occur in odontoblasts and ameloblasts (João and Arana‐Chavez, 2004[36]). With respect to TJs, different claudins are expressed at different stages from epithelial thickening to the onset of differentiation (Ohazama and Sharpe, 2007[57]). ZO-1 is the first protein to be detected in ameloblast and odontoblast layers in the early stages of odontogenesis (João and Arana-Chavez, 2003[35]). Ameloblasts encompass TJs at 2 locations, namely at the proximal part, which is in the vicinity of the stratum intermedium, and at the distal part in the secretory pole. On the contrary, odontoblasts only have TJs at their distal area. Moreover, the structure of TJs is macular between odontoblasts, while ameloblasts have zonula TJs. It has been suggested that ZO-1 is more related to the generation and preservation of cellular polarization, whereas claudins and occludin form barriers that restrict the passage of substances to the early developing odontoblasts and ameloblasts (João and Arana‐Chavez, 2004[36]). 

TJs are involved in carcinogenesis in the oral cavity, including oral squamous cell carcinoma (OSCC). The loss of TJ proteins can induce dedifferentiation and promotes cancer progression. This is in line with the findings of several studies showing carcinogenesis, tumor recurrence and poor survival in patients with loss of TJ molecules in different cancer types (Martin et al., 2010[51]). However, other studies show that overexpression of TJ proteins, in particular JAM and claudins, is linked to tumor growth. Hence, it has been suggested that increased TJ protein expression, rather than their loss, facilitates carcinogenesis (Leech et al., 2015[45]). Overexpression of claudin-1 has been observed in advanced stages of OSCC, coinciding with angiolymphatic and perineural tumor invasion (dos Reis et al., 2008[21]). This is associated with activation of matrix metalloproteases (MMPs) and therefore increased cleavage of extracellular matrix components. Moreover, the activation of MMPs and OSCC invasion become more manifested upon suppression of claudin-1 production (Oku et al., 2006[59]). CAR also has a critical role in the progression of OSCC by promoting cancer cell growth and survival, while negatively affecting the apoptotic machinery. The latter is mediated *via* the specific interaction of CAR with Rho-associated protein kinase and its subsequent inhibition that accelerates cell-cell adhesion required for cancer cell growth. OSCC growth is reduced because of cell dissociation in the absence of CAR expression (Saito et al., 2014[67]). 

## Gap Junctions

### General features of gap junctions

**Figure 2:**
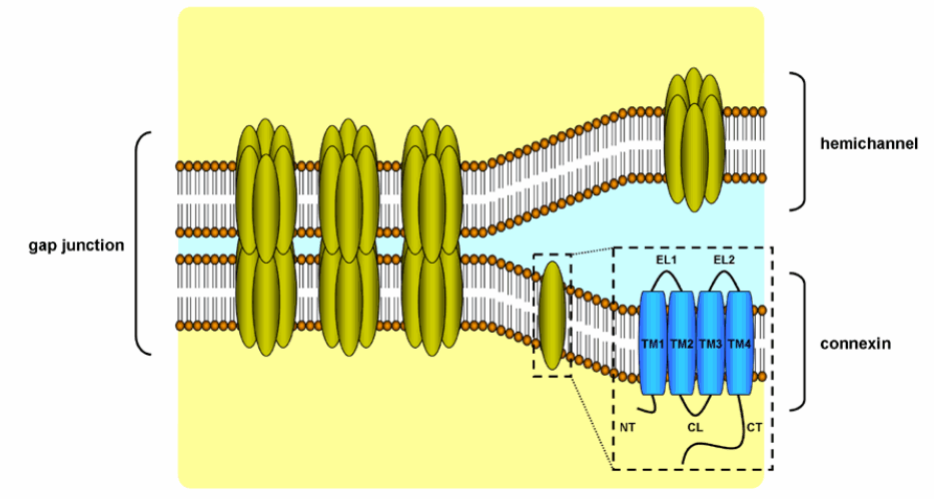
Structure of gap junctions. Gap junctions located at the cell plasma membrane are built up by 2 hemichannels that each contain 6 connexin proteins. Connexin proteins consist of 4 transmembrane domains (TM1-4), one cytoplasmic loop (CL), 2 extracellular loops (EL), one cytoplasmic C-terminal tail (CT) and one cytoplasmic N-terminal tail.

GJs provide a pathway for direct communication between adjacent cells (Scott and Kelsell, 2011[70]). GJs enable the intercellular transfer of small and hydrophilic substances. GJs appear as hexagonal arrays at the separation of 2 neighboring cells in electron microscopy or X-ray crystallography. A plethora of factors regulates GJ opening and closing, including pH, calcium concentration and posttranslational modifications (Wei et al., 2004[79]). Like TJs, GJs have a dynamic structure, yet they do not directly bind to the cytoskeleton. GJs are composed of hemichannels, also called connexons, of adjacent cells, which in turn are built up by 6 connexin (Cx) proteins (Figure 2(Fig. 2)) (Table 1(Tab. 1)) (Ahmadian et al., 2019[3]). Hemichannels can be either homomeric or heteromeric depending on the identity of the Cx subunits. As much as 21 Cx proteins have been identified in humans. They are named according to their molecular weight expressed in kilodalton. Connexin proteins all have an identical structure, consisting of 4 transmembrane domains, 2 extracellular loops, a cytoplasmic loop, a cytosolic C-terminal tail and a N-terminal tail. Assembly, trafficking and channel gating properties of GJs are largely mediated by phosphorylation mainly occurring at the C-terminus of Cx proteins (Decrock et al., 2009[20]). Coupling and communication *via* GJs are closely related to cadherin-based AJs, since inhibition of AJs disturbs GJ formation and function (Wan et al., 2018[78]). 

GJs are found in the basal, spinous and granular layers of the epidermis in the oral mucosa. Different Cx isoforms have been detected in the epidermis of oral mucosa at different phases of keratinocyte differentiation, suggesting distinct roles. Cx43, Cx32, Cx30, and Cx26 are expressed in gingival epithelial cells and buccal mucosa of mice. Electron microscopy and freeze-fracture experiments have shown the presence of GJs between odontoblasts and sub-odontoblastic cells. Cx43 is expressed in the developing rodent teeth and correlates with the extent of differentiation (Fried et al., 2003[27]). Although detected in humans, Cx43 is downregulated in permanent adult teeth and seems to be replaced by Cx26 (Fried et al., 2003[27]). 

Cx43 is highly expressed in human fibroblasts. Cx32 and Cx43 are the predominant Cx proteins found in human periodontal ligament fibroblasts (Yamaoka et al., 2000[82]). The fibrous connective tissue structure of dental pulp contains fibroblasts, odontoblasts and undifferentiated cells around dentin. Fibroblasts are considered as key components of the dental pulp, since they generate collagen, collagenase and proteoglycan. Moreover, they have the capacity to differentiate into odontoblasts, while primary odontoblasts are impaired. Cx43, Cx32 and Cx26 are expressed in human dental pulp fibroblasts (Ibuki et al., 2002[34]). 

### Gap junctions and oral health

Normal epithelial cells show Cx43 and Cx26 production, whereas OSCC cells only express Cx43 (Frank et al., 2006[26]). In fact, Cx43 has been proposed as a prognostic biomarker in OSCC associated with poor survival (Brockmeyer et al., 2014[12]). GJs exert different functions depending on the OSCC stage. Cx proteins regulate cell cycling by affecting the transcription of genes coding for cyclins and cyclin-dependent kinases (Cronier et al., 2008[17]). The loss of GJs typically enhances cell proliferation. Cx proteins may promote attachment of cancerous cells to the stroma during tumor metastasis. Cx26 overexpression has been detected in tissue specimens of OSCC and lymph node metastasis (Villaret et al., 2000[75]). Unlike Cx43, Cx26 and Cx45 have been reported to have no prognostic value in OSCC (Brockmeyer et al., 2014[12]).

Dental caries occurs in the presence of micro-organisms and leads to decalcification and proteolysis of dentin. Odontoblasts become active secretory resources and tubuli are calcified during dental caries (Tziafas et al., 2000[74]). While odontoblasts surround the carious lesion, Cx43 expression is increased in adult dental tissue, thus suggesting a role for GJs in hypercalcification. It has been shown that cultivation of human pulp cells can trigger the production of dentin (About et al., 2000[1]). Dental pulp cells express low amounts of Cx43 and mineralization processes enhance its expression. This is in line with the observation that calcium levels in the extracellular matrix surrounding osteoblast-like cells increase upon transfection with Cx43 cDNA (Gramsch et al., 2001[30]). These data substantiate the role of Cx43 in mineralization. This is further evidenced by the results of a number of studies showing that Cx43 deficiency is associated with delayed ossification, osteoblast malfunction and craniofacial abnormalities (Rossello and Kohn, 2009[66]). Osteogenesis and the activity of osteoblasts depend on the function of Cx43, since Cx43-null mice show diminished expression of specific genes involved in mineralization (Lecanda et al., 2000[44]). These data underscore the critical role of Cx43 in the secretory activity of odontoblasts during dentin mineralization, either in the stages of teeth development or during dental injuries. The increased expression of Cx43 in osteoclasts and periodontal ligament cells in the compression zone has been demonstrated in an orthodontic force model. Furthermore, the tension zones of the periodontal ligament show increased Cx43 levels in osteoblasts and osteocytes. Therefore, coordination of alveolar bone remodeling might be depending on the activity of Cx43. Moreover, the synchronization of odontoblasts with mineral matrix deposition is mediated by Cx43 in human teeth (About et al., 2002[2]). 

Maxillofacial surgeries or extraction of mandibular molars can cause injuries in the mandibular nerve, which results in chronic orofacial pain. Innervation of this region with trigeminal nerve branches can spread the pain to the adjacent orofacial regions (Renton et al., 2012[64]). Using an *in vivo* model, the mechanism of ectopic orofacial pain in mandibular nerve *via* transection of the inferior alveolar nerve has been investigated, thereby developing pain hypersensitivity in the whisker pad skin (Okada-Ogawa et al., 2015[58]). Intra-trigeminal ganglion-mediated pain transmission plays a crucial role in mediating trigeminal nociceptive mechanisms. Complete wrapping of satellite glial cells (SGCs) around the soma of sensory neurons occurs in the trigeminal ganglion. The presence of glial fibrillary acidic protein in SGCs distinguishes them from other neural cells (Hanani, 2005[32]). However, this protein is significantly upregulated in pathological conditions, such as cancer, inflammation and peripheral nerve injury (Hanani, 2005[32]). Couplings of SGC cells is directly related to the number of GJs during peripheral nerve injury (Vit et al., 2007[76]). Furthermore, Cx43 expression increases in the trigeminal nerve following chronic constriction injury of the infraorbital nerve (Kaji et al., 2016[37]). 

Mutations in Cx genes have been linked to several human diseases, such as hearing loss, skin disease and dental problems (Krutovskikh and Yamasaki, 2000[43]). Although the exact mechanisms by which these mutations result in disease are not entirely clear, the accumulation of Cx mutant proteins in the cytoplasm, loss of GJ activity and aberrant hemichannel functioning may be involved (Scott and Kelsell, 2011[70]). Oculodentodigital dysplasia (ODDD) is an unusual autosomal dominant disorder typified by ocular, craniofacial, dental and digital abnormalities. Enamel hypoplasia, anodontia and premature loss of teeth are common dental problems in ODDD. Several dominant mutations in the Cx43 gene have been observed in ODDD (de la Parra and Zenteno, 2007[19]). 

## Anchoring Junctions

### General features of anchoring junctions

**Figure 3:**
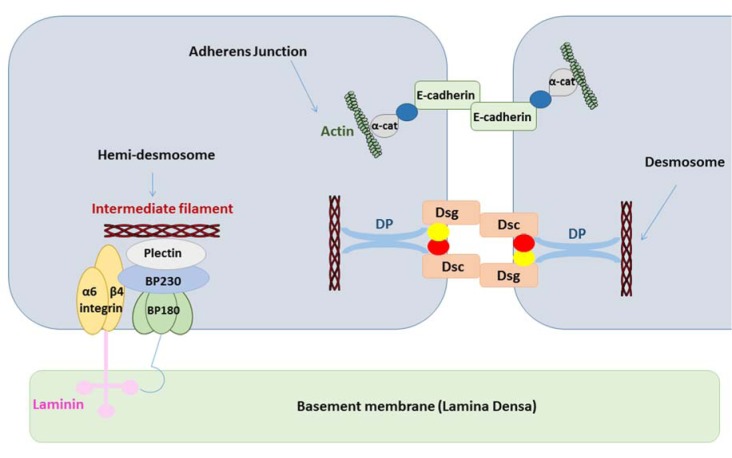
Structure of anchoring junctions. Adherens junctions are attached to actin, while desmosomes and hemi-desmosomes are linked to intermediate filaments (α-cat, α-catenin; BP180/230, bullous pemphigoid 180/230; DP, desmoplakin; Dsc, desmocollin; Dsg, desmoglein)

Anchoring junctions consist of AJs, desmosomes and hemi-desmosomes, and are key structures in the maintenance of tissue architecture. AJs are coupled with the cytoskeleton *via* catenins and mediate the assembly of actin. Desmosomes rather bind to the intermediate filament cytoskeleton. Classical cadherins, such as E-cadherin, form the transmembrane core of AJs (Figure 3(Fig. 3)) (Table 1(Tab. 1)). The ectodomain of cadherin binds calcium and regulates trans-oligomerization between cadherins of neighboring cells. The extracellular domain of cadherins encompasses 5 motifs that are separated by bendable hinges. The binding of calcium in the hinge juxtaposition prevents its flexing. Thus, a rigid and curved shape is formed in the extracellular domain (Wan et al., 2018[77]). The link between the N-terminal domains of cadherins from 2 opposing cells generates cell adhesion. Cadherins are attached to actin at the cytosolic side *via* catenins. The stabilization of cadherin clusters is mediated by armadillo proteins, including α-catenin and β-catenin. AJs are considered as key elements of tissue morphogenesis and homeostasis, since they support tissue dynamics and structural organization (Bierbaumer et al., 2018[8]).

Desmosomes form an architectural system by linking to intermediate filaments in order to preserve the mechanical strength of the epithelium. Cellular differentiation is regulated, at least in part, by desmosomes, which act as surface receptors that mediate intercellular communication. Both the cell type and differentiation status determine desmosomal composition. Mutations in desmosomal cadherins lead to anomalous proliferation and differentiation (Garrod and Chidgey, 2008[28]). Thus, the ectopic expression of desmosomal proteins enhances downstream epidermal growth factor receptor signaling, resulting in suppression of epidermis differentiation and induction of papilloma formation (Brennan et al., 2007[11]). Desmosomes are composed of several protein families, including desmogleins (Dsgs), desmocollins (Dscs), armadillo proteins, including plakoglobin and plakophilin, and plankin proteins, such as desmoplakin (DP). Dsg and Dsc proteins regulate direct adhesion of neighboring cells, while armadillo proteins are linked with the cytoplasmic domain of cadherins. Plankin proteins anchor stress-bearing intermediate filaments to desmosomes (North et al., 1999[56]). 

Hemi-desmosomes connect the intermediate filament network of epithelial cells to the underlying basement membrane in different tissues, including the oral cavity, pharynx and larynx. Hemi-desmosomes appear as half of a desmosome and contain a triple electron-dense plaque architecture, known as inner, outer and sub-basal hemi-desmosomal plaques (Borradori and Sonnenberg, 1999[9]). Plectin, as a plakin family protein, is a major component of hemi-desmosomes and consists of a central coiled-coil rod domain adjoined with a N-terminal head and a C-terminal tail (Koster et al., 2004[42]). The attachment of plectin to intermediate keratin filaments is mediated through bullous pemphigoid 230 (BP230) (Koster et al., 2004[42]). BP230 is also a plakin protein family with a structure reminiscent of plectin (Koster et al., 2003[41]). Collagen XVII, which was formerly known as BP180, is a transmembrane hemi-desmosomal glycoprotein with an intracellular non-collagenous N-terminal domain and an extracellular C-terminus, encompassing collagen repeats that bind to laminin (Hashmi and Marinkovich, 2011[33]). Different tissues, such as skin, mucus and teeth, express BP180. The core of hemi-desmosomes is a transmembrane protein called integrin α6β4. The larger intracellular domain of integrin, namely the β4 subunit, interacts with the intracellular part of collagen XVII, which in turn links the intermediate keratin filaments with BP230 and plectin, while the extracellular domain of integrin α6β4 is a receptor for laminin (Borradori and Sonnenberg, 1999[9]). 

### Anchoring junctions and oral health

Any disturbance in the function of cadherin-consisting AJs results in several human pathologies, such as cancer, inflammation and auto-immune disorders (Wheelock and Johnson, 2003[81]; Garrod et al., 2002[29]). Aberrant expression of E-cadherin is associated with tumor metastasis and invasiveness, and might serve as a prominent prognostic marker in cancer (Moh and Shen, 2009[55]).

Periodontal disease constitutes a prevalent health problem worldwide (Cullinan and Seymour, 2013[18]). Periodontitis and gingivitis are classified as infectious periodontal diseases, and can be either acute or chronic (Pihlstrom et al., 2005[62]). The infection in periodontitis destroys the supporting periodontal tissue through the release of proteolytic enzymes and inflammatory cytokines from immune cells (Ebersole et al., 2013[22]). The pocket formation, inflammation and the activity of osteoclasts are hallmarks of periodontitis and highly depend on cell-cell junctions (Wan et al., 2018[78]). 

Failure of the teeth to attach to the epithelium results in the generation of a pocket or cleft, which acts as a rate-limiting barrier for penetration of microbial products into tissues. It has been suggested that developmental remnants of the junctional epithelium are the sources of non-keratinizing squamous epithelial cells in the pocket. The aberrant morphological structure of the pocket has been associated with the lack of a clear layered assembly. E-cadherin is considered a prominent mediator in the maintenance of tissue integrity in squamous epithelia. The morphology and function of adherens junction and desmosomal proteins are preserved through the function of E-cadherin (Wheelock and Jensen, 1992[80]). 

Desmosomes mediate different signaling pathways involved in cell proliferation, differentiation and morphogenesis. Deregulation of these signaling pathways may trigger various diseases. In this respect, bacterial proteases of pathogenic bacteria, such as *Staphylococcus aureus*, target Dsg1, leading to blister formation in infectious diseases (Amagai et al., 2000[4]). Acantholysis, the loss of keratinocyte adhesion in the spinous layer of the epithelium, is a common clinical manifestation of pemphigus in the skin and oral mucosa. The presence of auto-antibodies directed against Dsg1 causes the generation of blisters in the upper granular layers of the epidermis in *pemphigus foliaceus*. *Pemphigus vulgaris* is associated with oral lesions and is accompanied by the production of Dsg3 auto-antibodies (Wan et al., 2018[77]). 

Oral *lichen planus*, which is characterized by an epidermal auto-immune attack, is considered as an idiopathic inflammatory disorder. Chronic immune damage to keratinocytes located in the oral submucosa is thought to play a key role in this disease (Thornhill, 2001[73]). High concentrations of auto-antibodies against Dsg1 and Dsg3 have been observed in erosive *lichen planus* patients (Lukač et al., 2006[48]). Furthermore, it has been reported that disruption of hemi-desmosomes results in the degeneration of basal keratinocytes and the deregulation of basal membranes, which produces weakness in epithelial connective tissues of the oral cavity in *lichen planus* (Lucchese, 2015[47]). 

Mucosal pemphigoid, an auto-immune blistering disorder, is caused by the formation of auto-antibodies against the structural components of hemi-desmosomes and/or basement membrane zone (Scully and Muzio, 2008[71]). The generated auto-antibodies deregulate the binding of basal keratinocytes to the underlying basement membrane, leading to the occurrence of sub-epithelial blisters (Schmidt and Zillikens, 2013[69]). Auto-antibodies against integrin α6β4, BP180, BP230, laminin and their subunits might be produced (Feller et al., 2017[24]). This disease is commonly initiated in the mouth and is occasionally restricted to the oral mucosa (Kasperkiewicz et al., 2012[38]). The rupture of sub-epithelial blisters results in the rapid formation of fibrinous pseudomembranes around the painful ulcers. Gingiva, palate and buccal mucosa are the most frequently affected parts in the oral cavity. Desquamative gingivitis, which is the gingival form of mucosal pemphigoid, is often the only clinical manifestation of the disease (Lo Russo et al., 2008[46]). The reactive antibodies against hemi-desmosomal components might also fix complement constituents, mediating an inflammatory process that further promotes the detachment of basal cells from the basement membrane (Chan, 2012[15]).

## Conclusions

Cell-cell and cell-extracellular matrix junctions play a pivotal role in tissue integrity, repair systems and homeostasis. Not surprisingly, their disruption underlies a wide range of human disorders, such as inflammation, cancer, auto-immune and hereditary diseases. The oral cavity and its appendices express several types of junctional proteins that act as key components of developmental processes in oral epithelium, dental structures and salivary glands. It has been shown that the structure and functionality of cell-cell and cell-extracellular matrix junctions are altered in oral cavity-related diseases, yet further in-depth investigation is required. In this context, recent improvements in tissue culture methods and bio-engineering techniques as well as the availability of knock-out animal models for junctional proteins will allow unveiling yet unknown functions of cell-cell and cell-extracellular matrix junctions in the oral cavity. This may open perspectives for the establishment of new clinical strategies to treat diseases related to the oral cavity. 

## Notes

Mohammad Samiei and Elham Ahmadian contributed equally as first authors.

## Acknowledgements

The authors are thankful to the Tabriz Medical University's “Students' Research Committee” and Maragheh University of Medical Sciences for the support of this manuscript.

## Conflict of interest

The authors declare that there are no conflicts of interest associated with this work.

## Figures and Tables

**Table 1:**
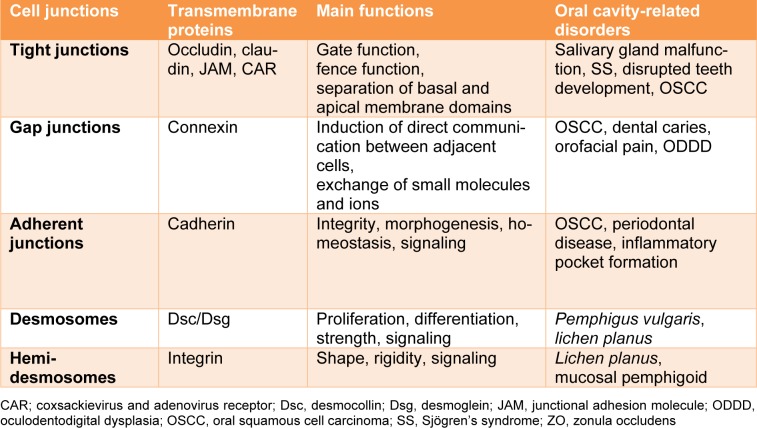
Cell-cell and cell-extracellular matrix junctions in the oral cavity

## References

[1] About I, Bottero M-J, de Denato P, Camps J, Franquin J-C, Mitsiadis TA (2000). Human dentin production in vitro. Exp Cell Res.

[2] About I, Proust J-P, Raffo S, Mitsiadis TA, Franquin J-C (2002). In vivo and in vitro expression of connexin 43 in human teeth. Connec Tissue Res.

[3] Ahmadian E, Eftekhari A, Samiei M, Maleki Dizaj S, Vinken M (2019). The role and therapeutic potential of connexins, pannexins and their channels in Parkinson's disease. Cell Signal.

[4] Amagai M, Matsuyoshi N, Wang ZH, Andl C, Stanley JR (2000). Toxin in bullous impetigo and staphylococcal scalded-skin syndrome targets desmoglein 1. Nat Med.

[5] Anderson JM (2001). Molecular structure of tight junctions and their role in epithelial transport. Physiology.

[6] Baker OJ (2010). Tight junctions in salivary epithelium. Biomed Res Int.

[7] Baker OJ, Camden JM, Redman RS, Jones JE, Seye CI, Erb L (2008). Proinflammatory cytokines tumor necrosis factor-α and interferon-γ alter tight junction structure and function in the rat parotid gland Par-C10 cell line. Am J Physiol.

[8] Bierbaumer L, Schwarze UY, Gruber R, Neuhaus W (2018). Cell culture models of oral mucosal barriers: A review with a focus on applications, culture conditions and barrier properties. Tissue Barriers.

[9] Borradori L, Sonnenberg A (1999). Structure and function of hemidesmosomes: more than simple adhesion complexes. J Invest Dermatol.

[10] Bosveld F, Markova O, Guirao B, Martin C, Wang Z, Pierre A (2016). Epithelial tricellular junctions act as interphase cell shape sensors to orient mitosis. Nature.

[11] Brennan D, Hu Y, Joubeh S, Choi YW, Whitaker-Menezes D, O'Brien T (2007). Suprabasal Dsg2 expression in transgenic mouse skin confers a hyperproliferative and apoptosis-resistant phenotype to keratinocytes. J Cell Science.

[12] Brockmeyer P, Jung K, Perske C, Schliephake H, Hemmerlein B (2014). Membrane connexin 43 acts as an independent prognostic marker in oral squamous cell carcinoma. Int J Oncol.

[13] Carpenter GH (2013). The secretion, components, and properties of saliva. Annu Rev Food Sci Technol.

[14] Catalán MA, Nakamoto T, Melvin JE (2009). The salivary gland fluid secretion mechanism. J Med Invest.

[15] Chan LS (2012). Ocular and oral mucous membrane pemphigoid (cicatricial pemphigoid). Clin Dermatol.

[16] Coyne CB, Bergelson JM (2005). CAR: a virus receptor within the tight junction. Adv Drug Deliv Rev.

[17] Cronier L, Crespin S, Strale P-O, Defamie N, Mesnil M (2008). Gap junctions and cancer: new functions for an old story. Antioxid Redox Signal.

[18] Cullinan MP, Seymour GJ (2013). Periodontal disease and systemic illness: will the evidence ever be enough?. Periodontology.

[19] de la Parra DR, Zenteno JC (2007). A new GJA1 (Connexin 43) mutation causing oculodentodigital dysplasia associated to uncommon features. Ophthalmic Genet.

[20] Decrock E, Vinken M, De Vuyst E, Krysko DV, D'Herde K, Vanhaecke T (2009). Connexin-related signaling in cell death: to live or let die?. Cell Death Differ.

[21] dos Reis PP, Bharadwaj RR, Machado J, MacMillan C, Pintilie M, Sukhai MA (2008). Claudin 1 overexpression increases invasion and is associated with aggressive histological features in oral squamous cell carcinoma. Cancer.

[22] Ebersole JL, Dawson DR, Morford LA, Peyyala R, Miller CS, Gonzaléz OA (2013). Periodontal disease immunology:‘double indemnity’in protecting the host. Periodontology.

[23] Ewert P, Aguilera S, Alliende C, Kwon Y-J, Albornoz A, Molina C (2010). Disruption of tight junction structure in salivary glands from Sjögren's syndrome patients is linked to proinflammatory cytokine exposure. Arthritis Rheumatol.

[24] Feller L, Ballyram R, Khammissa R, Altini M, Lemmer J (2017). Immunopathogenic oral diseases: an overview focusing on pemphigus vulgaris and mucous membrane pemphigoid. Oral Health Prev Dent.

[25] Fox RI, Kang HI, Ando D, Abrams J, Pisa E (1994). Cytokine mRNA expression in salivary gland biopsies of Sjögren's syndrome. J Immunol.

[26] Frank DK, Szymkowiak B, Hughes CA (2006). Connexin expression and gap junctional intercellular communication in human squamous cell carcinoma of the head and neck. Otolaryngol Head Neck Surg.

[27] Fried K, Mitsiadis T, Guerrier A, Haegerstrand A, Meister B (2003). Combinatorial expression patterns of the connexins 26, 32, and 43 during development, homeostasis, and regeneration of rat teeth. Int J Dev Biol.

[28] Garrod D, Chidgey M (2008). Desmosome structure, composition and function. Biochim Biophys Acta Biomembr.

[29] Garrod DR, Merritt AJ, Nie Z (2002). Desmosomal cadherins. Curr Opin Cell Biol.

[30] Gramsch B, Gabriel H-D, Wiemann M, Grümmer R, Winterhager E, Bingmann D (2001). Enhancement of connexin 43 expression increases proliferation and differentiation of an osteoblast-like cell line. Exp Cell Res.

[31] Groeger SE, Meyle J (2015). Epithelial barrier and oral bacterial infection. Periodontology 2000.

[32] Hanani M (2005). Satellite glial cells in sensory ganglia: from form to function. Brain Res Rev.

[33] Hashmi S, Marinkovich MP (2011). Molecular organization of the basement membrane zone. Clin Dermatol.

[34] Ibuki N, Yamaoka Y, Sawa Y, Kawasaki T, Yoshida S (2002). Different expressions of connexin 43 and 32 in the fibroblasts of human dental pulp. Tissue Cell.

[35] João SM, Arana-Chavez VE (2003). Expression of connexin 43 and ZO-1 in differentiating ameloblasts and odontoblasts from rat molar tooth germs. Histochem Cell Biol Title.

[36] João SM, Arana‐Chavez VE (2004). Tight junctions in differentiating ameloblasts and odontoblasts differentially express ZO‐1, occludin, and claudin‐1 in early odontogenesis of rat molars. Anat Rec A Discov Mol Cell Evol Biol.

[37] Kaji K, Shinoda M, Honda K, Unno S, Shimizu N, Iwata K (2016). Connexin 43 contributes to ectopic orofacial pain following inferior alveolar nerve injury. Mol Pain.

[38] Kasperkiewicz M, Zillikens D, Schmidt E (2012). Pemphigoid diseases: pathogenesis, diagnosis, and treatment. Autoimmunity.

[39] Kawedia JD, Nieman ML, Boivin GP, Melvin JE, Kikuchi K-I, Hand AR (2007). Interaction between transcellular and paracellular water transport pathways through Aquaporin 5 and the tight junction complex. Proc Natl Acad Sci USA.

[40] Khalili A, Ahmad M (2015). A review of cell adhesion studies for biomedical and biological applications. Int J Mol Sci.

[41] Koster J, Geerts D, Favre B, Borradori L, Sonnenberg A (2003). Analysis of the interactions between BP180, BP230, plectin and the integrin α6β4 important for hemidesmosome assembly. J Cell Sci.

[42] Koster J, Van Wilpe S, Kuikman I, Litjens S, Sonnenberg A (2004). Role of binding of plectin to the integrin β4 subunit in the assembly of hemidesmosomes. Mol Biol Cell.

[43] Krutovskikh V, Yamasaki H (2000). Connexin gene mutations in human genetic diseases. Mutat Res Rev Mutat Res.

[44] Lecanda F, Warlow PM, Sheikh S, Furlan F, Steinberg TH, Civitelli R (2000). Connexin43 deficiency causes delayed ossification, craniofacial abnormalities, and osteoblast dysfunction. J Cell Biol.

[45] Leech AO, Cruz RG, Hill AD, Hopkins AM (2015). Paradigms lost—an emerging role for over-expression of tight junction adhesion proteins in cancer pathogenesis. Ann Transl Med.

[46] Lo Russo L, Fedele S, Guiglia R, Ciavarella D, Lo Muzio L, Gallo P (2008). Diagnostic pathways and clinical significance of desquamative gingivitis. J Periodontol.

[47] Lucchese A (2015). A potential peptide pathway from viruses to oral lichen planus. J Med Virol.

[48] Lukač J, Brozović S, Vučićević-Boras V, Mravak-Stipetić M, Malenica B, Kusić Z (2006). Serum autoantibodies to desmogleins 1 and 3 in patients with oral lichen planus. Croatian Med J.

[49] Mandell KJ, Parkos CA (2005). The JAM family of proteins. Adv Drug Deliv Rev.

[50] Markov AG, Aschenbach JR, Amasheh S (2015). Claudin clusters as determinants of epithelial barrier function. IUBMB Life.

[51] Martin TA, Mansel RE, Jiang WG (2010). Loss of occludin leads to the progression of human breast cancer. Int J Mol Med.

[52] Masedunskas A, Sramkova M, Parente L, Sales KU, Amornphimoltham P, Bugge TH (2011). Role for the actomyosin complex in regulated exocytosis revealed by intravital microscopy. Proc Natl Acad Sci USA.

[53] Maślińska M, Przygodzka M, Kwiatkowska B, Sikorska-Siudek K (2015). Sjögren’s syndrome: still not fully understood disease. Rheumatol Int.

[54] Matter K, Balda MS (2003). Signalling to and from tight junctions. Nat Rev Mol Cell Biol.

[55] Moh MC, Shen S (2009). The roles of cell adhesion molecules in tumor suppression and cell migration. Cell Adh Migr.

[56] North AJ, Bardsley WG, Hyam J, Bornslaeger EA, Cordingley HC, Trinnaman B (1999). Molecular map of the desmosomal plaque. J Cell Sci.

[57] Ohazama A, Sharpe PT (2007). Expression of claudins in murine tooth development. Dev Dyn.

[58] Okada-Ogawa A, Nakaya Y, Imamura Y, Kobayashi M, Shinoda M, Kita K (2015). Involvement of medullary GABAergic system in extraterritorial neuropathic pain mechanisms associated with inferior alveolar nerve transection. Exp Neurol.

[59] Oku N, Sasabe E, Ueta E, Yamamoto T, Osaki T (2006). Tight junction protein claudin-1 enhances the invasive activity of oral squamous cell carcinoma cells by promoting cleavage of laminin-5 γ2 chain via matrix metalloproteinase (MMP)-2 and membrane-type MMP-1. Cancer Res.

[60] Palacios J, Benito N, Berraquero R, Pizarro A, Cano A, Gamallo C (2004). Differential spatiotemporal expression of E-and P-cadherin during mouse tooth development. Int J Dev Biol.

[61] Peppi M, Ghabriel M (2004). Tissue‐specific expression of the tight junction proteins claudins and occludin in the rat salivary glands. J Anat.

[62] Pihlstrom BL, Michalowicz BS, Johnson NW (2005). Periodontal diseases. Lancet.

[63] Rehder D, Iden S, Nasdala I, Wegener J, Zu Brickwedde M-KM, Vestweber D (2006). Junctional adhesion molecule-a participates in the formation of apico-basal polarity through different domains. Exp Cell Res.

[64] Renton T, Yilmaz Z, Gaballah K (2012). Evaluation of trigeminal nerve injuries in relation to third molar surgery in a prospective patient cohort. Recommendations for prevention. Int J Oral Maxillofac Surg.

[65] Rosenthal R, Milatz S, Krug SM, Oelrich B, Schulzke J-D, Amasheh S (2010). Claudin-2, a component of the tight junction, forms a paracellular water channel. J Cell Sci.

[66] Rossello RA, Kohn DH (2009). Gap junction intercellular communication: a review of a potential platform to modulate craniofacial tissue engineering. J Biomed Mater Res B Appl Biomater.

[67] Saito K, Sakaguchi M, Iioka H, Matsui M, Nakanishi H, Huh N (2014). Coxsackie and adenovirus receptor is a critical regulator for the survival and growth of oral squamous carcinoma cells. Oncogene.

[68] Sakakibara A, Furuse M, Saitou M, Ando-Akatsuka Y, Tsukita S (1997). Possible involvement of phosphorylation of occludin in tight junction formation. J Cell Biol.

[69] Schmidt E, Zillikens D (2013). Pemphigoid diseases. Lancet.

[70] Scott CA, Kelsell DP (2011). Key functions for gap junctions in skin and hearing. Biochem J.

[71] Scully C, Muzio LL (2008). Oral mucosal diseases: mucous membrane pemphigoid. Br J Oral Maxillofac Surg.

[72] Shin K, Fogg VC, Margolis B (2006). Tight junctions and cell polarity. Annu Rev Cell Dev Biol.

[73] Thornhill M (2001). Immune mechanisms in oral lichen planus. Acta Odontol Scand.

[74] Tziafas D, Smith A, Lesot H (2000). Designing new treatment strategies in vital pulp therapy. J Dent.

[75] Villaret DB, Wang T, Dillon D, Xu J, Sivam D, Cheever MA (2000). Identification of genes overexpressed in head and neck squamous cell carcinoma using a combination of complementary dna subtraction and microarray analysis. Laryngoscope.

[76] Vit J-P, Jasmin L, Bhargava A, Ohara PT (2007). Satellite glial cells in the trigeminal ganglion as a determinant of orofacial neuropathic pain. Neuron Glia Biol.

[77] Wan H, Gadmor H, Brown L, Bergmeier LA (2018). Anchoring junctions in the oral mucosa: adherens junctions and desmosomes. Oral mucosa in health and disease: a concise handbook.

[78] Wan H, Gadmor H, Brown L, Bergmeier LA (2018). Cell-cell interactions in the oral mucosa: tight junctions and gap junctions. Oral mucosa in health and disease: a concise handbook.

[79] Wei C-J, Xu X, Lo CW (2004). Connexins and cell signaling in development and disease. Annu Rev Cell Dev Biol.

[80] Wheelock MJ, Jensen PJ (1992). Regulation of keratinocyte intercellular junction organization and epidermal morphogenesis by E-cadherin. J Cell Biol.

[81] Wheelock MJ, Johnson KR (2003). Cadherin-mediated cellular signaling. Curr Opin Cell Biol.

[82] Yamaoka Y, Sawa Y, Ebata N, Ibuki N, Yoshida S, Kawasaki T (2000). Double expressions of connexin 43 and 32 in human periodontal ligament fibroblasts. Tissue Cell.

